# Comparative Evaluation of the Accuracy of Two-Dimensional and Three-Dimensional Radiographic Assessment of Bony Defects Before and After Endodontic Surgery

**DOI:** 10.7759/cureus.101835

**Published:** 2026-01-19

**Authors:** Aishwarya Talakeri, Pravin Kumar, Soundharrajan P, Vinay Kumar Chugh, Rajat Sharma, Arun Patnana

**Affiliations:** 1 Conservative Dentistry and Endodontics, All India Institute of Medical Sciences, Jodhpur, Jodhpur, IND; 2 Orthodontics and Dentofacial Orthopaedics, All India Institute of Medical Sciences, Jodhpur, Jodhpur, IND; 3 Conservative Dentistry and Endodontics, Manav Rachna Dental College, Faridabad, IND; 4 Pedodontics and Preventive Dentistry, All India Institute of Medical Sciences, Rajkot, Rajkot, IND

**Keywords:** cbct, endodontic surgery, image j, itk-snap, periapical lesions

## Abstract

Aim: The aim of this study was to evaluate and compare the accuracy of two-dimensional (2D) and three-dimensional (3D) imaging for the assessment of healing in bony defects before and after endodontic surgery.

Methodology: A total of 92 patients with a history of dental trauma and associated periapical pathology involving the anterior and premolar teeth of the maxilla and mandible were included in the study. Preoperative imaging comprised 2D intraoral periapical radiographs (IOPARs) and 3D cone-beam computed tomography (CBCT) scans of the region of interest. Endodontic surgery was performed in all patients following prior root canal treatment (RCT), and the bony defects were managed using platelet-rich fibrin (PRF). At one-year follow-up, both 2D and 3D imaging were repeated. Lesion area on 2D IOPARs was measured using ImageJ software, whereas volumetric analysis was performed on CBCT images using ITK-SNAP.

Results: The mean percentage reduction in lesion area was 91.31% ± 10.14%, whereas the mean volumetric reduction was significantly lower at 78.27% ± 16.00%. Median values were 95.21% (interquartile range (IQR): 15.74%) for area reduction and 75.78% (IQR: 27.18%) for volume reduction. The ranges further highlighted this discrepancy, with area reductions spanning 65.14-100% and volume reductions spanning 37.08-100%.

Conclusion: While 2D IOPARs are convenient for routine evaluation, they overestimate periapical healing compared with 3D volumetric assessment. CBCT combined with ITK-SNAP provides a more accurate, objective, and clinically reliable evaluation of postoperative bone healing, thereby supporting evidence-based endodontic practice.

## Introduction

Nonsurgical root canal therapy is the first-line treatment for periapical pathologies, with long-term success rates of 80-85% [1]. However, complete resolution is not always achieved, even with contemporary instrumentation, irrigation, and obturation techniques. Persistent lesions may present clinically as pain, swelling, sinus tract formation, increased mobility, or radiographic evidence of unresolved pathology. When clinical symptoms persist or radiographic healing is incomplete despite technically adequate root canal therapy, surgical intervention becomes necessary [2].

Endodontic surgery, or endodontic microsurgery, extends beyond traditional apicoectomy by employing refined microsurgical techniques, enhanced visualization through a dental operating microscope, and biocompatible materials for root-end management [3]. Its primary objectives are to seal all portals of exit from the root canal system, remove infected or necrotic periapical tissues, and create conditions that allow functional regeneration of periapical tissues [4].

The prognosis of endodontic microsurgery is highly influenced by meticulous preoperative assessment and planning. Accurate imaging is critical for the comprehensive evaluation of lesion characteristics, including size, morphology, and proximity to surrounding anatomical structures. Furthermore, detailed analysis of root morphology, including the number of canals, curvature, and presence of apical delta or accessory canals, informs decisions regarding the extent of root resection and retrograde cavity preparation.

Intraoral periapical radiography is widely used to evaluate tooth anatomy, periapical pathology, root canal morphology, and prior treatments. However, as a two-dimensional (2D) technique, it has limitations such as anatomical superimposition, geometric distortion, and underestimation of lesion size, particularly in the buccolingual dimension. Studies suggest that intraoral periapical radiographs (IOPARs) may miss a substantial proportion of periapical lesions, potentially leading to an overestimation of treatment success [5]. Furthermore, conventional radiography exhibits lower diagnostic accuracy when precise spatial localization of complex dental and osseous pathology is required, whereas CBCT-based interpretations provide improved diagnostic reliability and three-dimensional (3D) spatial representation [6,7].

Cone-beam computed tomography (CBCT) has the potential to address the shortcomings of traditional 2D imaging techniques by providing three-dimensional visualization of teeth, periapical tissues, and adjacent anatomical structures [8]. This advanced imaging modality may facilitate the identification of clinically relevant details, including cortical perforations, accessory canals, and early bone loss [9]. Evidence-based guidelines from the European Society of Endodontology (ESE) recommend the use of CBCT for presurgical planning in complex cases and for postoperative evaluation when conventional radiographs are inconclusive [10].

While periapical radiography remains indispensable due to its accessibility and low radiation exposure, CBCT may offer enhanced diagnostic precision that is particularly valuable for surgical planning, preoperative assessment, and postoperative monitoring. Given the potential discrepancies between 2D and 3D imaging, understanding their relative accuracy in assessing bony defects is essential. Therefore, this study aims to evaluate and compare the accuracy of 2D and 3D imaging for the assessment of healing in bony defects before and after endodontic surgery, with the goal of refining postoperative monitoring and improving the predictability of surgical outcomes in endodontic practice.

## Materials and methods

This was a prospective observational study conducted at the Conservative Dentistry and Endodontics Unit, Department of Dentistry, All India Institute of Medical Sciences (AIIMS), Jodhpur, Rajasthan, India. Diagnostic imaging was performed in the Radiology Unit, while all endodontic surgical procedures were carried out in a dedicated minor surgery operatory under aseptic conditions. The study was approved by the Institutional Research Ethics Committee of AIIMS, Jodhpur (Ref No. AIIMS/IEC/2024/5079), and adhered to the principles of the Declaration of Helsinki. The study was conducted in accordance with the Strengthening the Reporting of Observational Studies in Epidemiology (STROBE) guidelines, and written informed consent was obtained from all participants after they were provided with detailed explanations of the study procedures and potential risks.

Sample size calculation

The sample size was determined based on healing proportions reported in previous studies by Schloss et al. [11] and Tanomaru-Filho et al. [12], which assessed periapical healing after endodontic surgery using 2D and 3D imaging. With a desired power of 80% and a significance level of 0.05, the minimum required sample size was calculated to be 92 participants.

Study population

Patients presenting to the outpatient department between April 2024 and November 2024 with clinical or radiographic signs suggestive of periapical pathology were screened.

Patients aged 18 years or older of any gender were included in the study if they presented with large periapical lesions of endodontic origin confirmed through clinical and radiographic examination. Eligible teeth were those requiring surgical intervention following unsuccessful nonsurgical root canal treatment. All participants were required to provide their willingness to participate in the study and be available for scheduled follow-up visits.

Patients were excluded if they had contraindications to endodontic surgery or systemic conditions known to adversely affect healing, including uncontrolled diabetes mellitus, bleeding disorders, or immunosuppression. Pregnant or lactating individuals were also excluded. Additionally, cases involving periapical lesions of non-endodontic origin were not considered for inclusion in the study.

Radiographic examination

All patients underwent standardized preoperative and postoperative imaging, including 2D digital IOPARs and 3D CBCT scans; 2D imaging was performed using the paralleling technique to minimize image distortion. IOPARs were acquired with an XIOS XG Supreme sensor (Dentsply Sirona Inc., Charlotte, North Carolina, United States) operating at 70 kV and 8 mA, with an exposure time of 90 ms, and images were captured and analyzed using Sidexis 4 software (Dentsply Sirona Inc.). A Planmeca CBCT unit operated at 90 kV and 9 mA, with a voxel size of 0.150 mm, a 5-second exposure time, and a field of view of 80 × 50 mm, was used to obtain 3D imaging. The CBCT volumes were reconstructed using Romexis software version 5.9.0 (Planmeca Oy), and axial, sagittal, and coronal sections were evaluated at a slice thickness of 0.07 mm with 1-mm intervals.

Measurement of bony defects

A single examiner calibrated for ImageJ (https://imagej.net/) and ITK-SNAP software (Penn Image Computing and Science Laboratory, University of Pennsylvania, Philadelphia, Pennsylvania, United States; https://www.itksnap.org/pmwiki/pmwiki.php) performed all measurements to minimize inter-observer variability.

**Figure 1:**
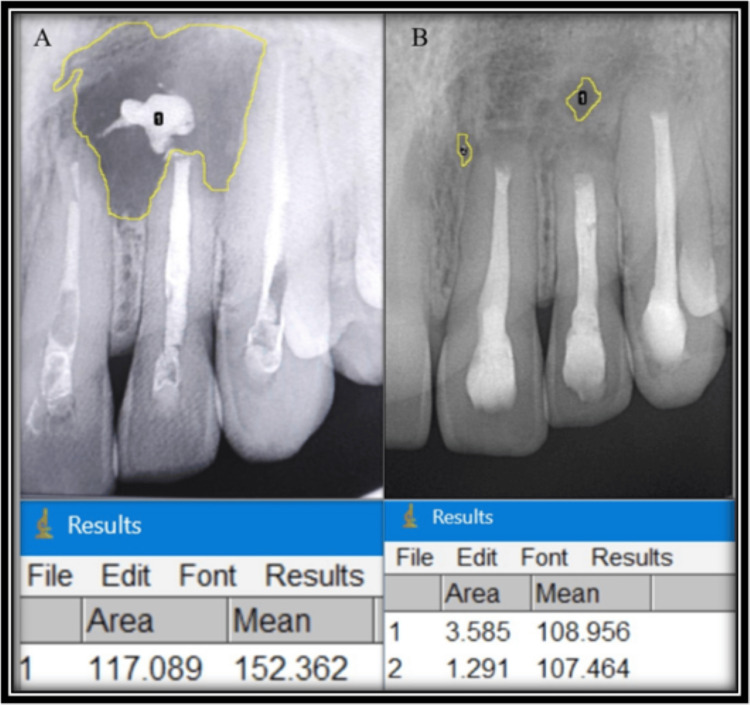
Preoperative (A) and one-year follow-up (B) analysis of 2D IOPARs using ImageJ software 2D: two-dimensional; IOPAR: intraoral periapical radiograph

A 2D lesion area analysis was performed using ImageJ software. After the images were imported into ImageJ, the software was calibrated using a ruler, with a 10-mm segment serving as the reference standard. This allowed accurate conversion of pixel measurements into millimeters, ensuring consistency across all participants. Following calibration, lesion boundaries were manually traced, and the lesion area was automatically calculated in square millimeters (mm²) (Figure 1).

**Figure 2:**
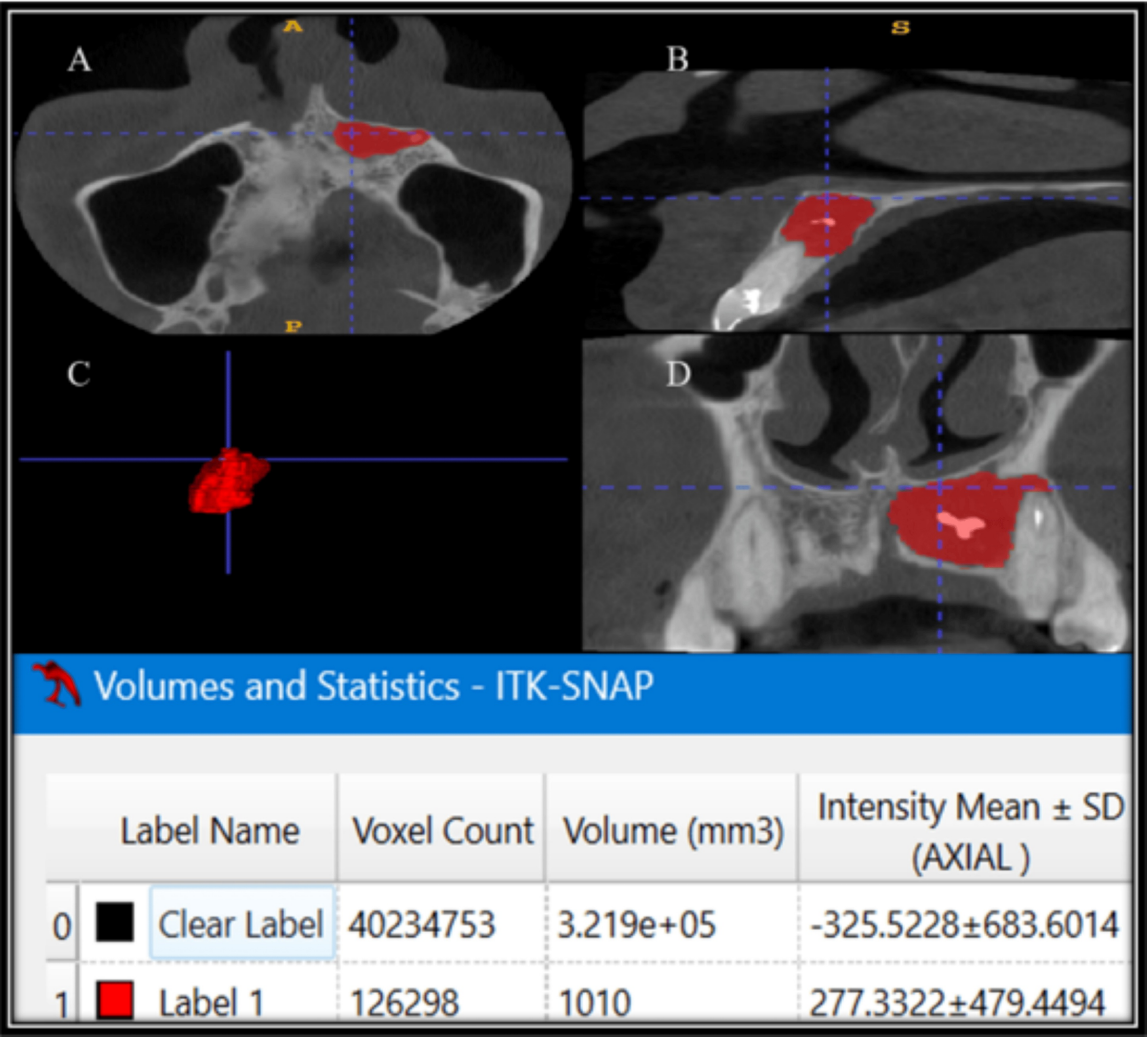
Preoperative volumetric analysis of 3D CBCT using ITK-SNAP software (A) Axial section, (B) Sagittal section, (C) 3D mesh, (D) Coronal section 3D: three-dimensional; CBCT: cone-beam computed tomography

**Figure 3:**
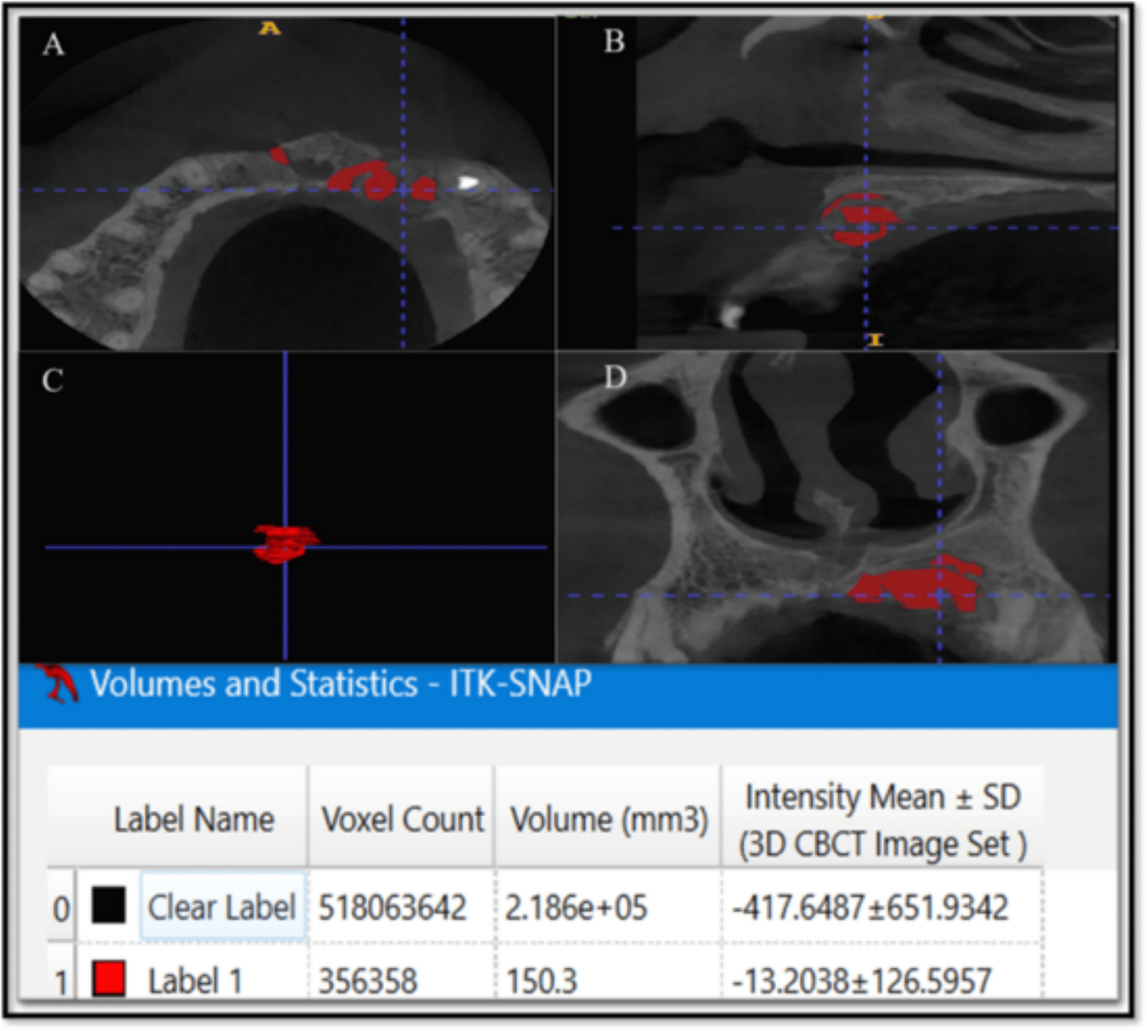
One-year follow-up volumetric analysis of 3D CBCT using ITK-SNAP software (A) Axial section, (B) Sagittal section, (C) 3D mesh, (D) Coronal section 3D: three-dimensional; CBCT: cone-beam computed tomography

A 3D volumetric analysis was conducted using ITK-SNAP software. CBCT images were imported into Digital Imaging and Communications in Medicine (DICOM) format, and voxel dimensions were verified prior to analysis. Lesions were segmented on a slice-by-slice basis using multiplanar views (axial, coronal, and sagittal), with semi-automatic region-growing tools followed by manual refinement to accurately delineate lesion boundaries. Segmentation was performed on all slices in which the lesion was visible to generate a contiguous three-dimensional reconstruction. The software automatically calculated lesion volume based on the number of segmented voxels and voxel size. Volumetric measurements were recorded in cubic millimeters (mm³) (Figures 2, 3).

Surgical procedure

Following completion of root canal treatment, endodontic surgery was performed under local anesthesia (2% lignocaine with 1:80,000 adrenaline). A submarginal scalloped rectangular or Luebke-Ochsenbein flap was elevated, and osteotomy was performed using a round carbide bur in a physiodispenser (W&H Implantmed Classic SI-923; W&H Dentalwerk Bürmoos GmbH, Bürmoos, Salzburg, Austria) at 40,000 rpm under copious irrigation. The pathological tissue was curetted and sent for histopathological examination.

Apical resection of 3 mm was performed perpendicular to the long axis, followed by retrograde cavity preparation using straight ultrasonic tips (F00106/F00079; Acteon Group, Mérignac, France). Retrograde cavities were filled incrementally with mineral trioxide aggregate (ProRoot MTA; Dentsply Maillefer, Ballaigues, Switzerland) and condensed using a microsurgical DE plugger. Platelet-rich fibrin (PRF) was placed in the bony defect, and the flap was repositioned and secured with 4-0 polytetrafluoroethylene (PTFE) sutures. A periodontal dressing (COE-PAK™ Automix; GC America Inc., Alsip, Illinois, United States) was applied, and postoperative IOPARs were obtained to assess the outcome of the procedure. Patients received antibiotics and analgesics for five days and were provided with standard postoperative care instructions.

Postoperative follow-up and imaging

Patients were recalled periodically, and at one year postoperatively, both 2D and 3D imaging were repeated under identical settings to allow a reliable comparison. Area and volumetric analyses of postoperative images were performed as for the preoperative images. The percentage reduction in lesion area (2D) and volume (3D) was calculated, and comparisons of 2D and 3D measurements were performed to evaluate differences in imaging accuracy.

Statistical analysis

Statistical analysis was performed using IBM SPSS Statistics for Windows, version 25.0 (IBM Corp., Armonk, New York, United States). Descriptive statistics for preoperative and postoperative area (mm²) and volume (mm³) included mean ± standard deviation (SD), median, and interquartile range (IQR), with graphical data representation. Normality of the data was assessed using the Shapiro-Wilk test (Table 1). Within-group (preoperative vs postoperative) comparisons were performed using the Wilcoxon signed-rank test. Intergroup comparisons (2D vs 3D) were performed using the Mann-Whitney U test. A significance level of p < 0.05 was considered statistically significant.

**Table 1:** Normality testing of preoperative and one-year postoperative area and volume measurements Normality of continuous variables was assessed using the Shapiro–Wilk test. All variables showed a non-normal distribution (p < 0.05). Therefore, nonparametric tests were used for further analysis: paired comparisons between preoperative and postoperative measurements were performed using the Wilcoxon signed-rank test, and unpaired comparisons between independent groups were performed using the Mann–Whitney U test. SD: standard deviation; IQR: interquartile range

Measurement	Mean	Median	SD	IQR	Minimum	Maximum	Shapiro-Wilk statistic	P value
Preoperative area (mm^2^)	91.862	66.045	81.227	83.659	7.914	483.178	0.791	< 0.01
Postoperative area (mm^2^)	9.440	3.992	15.638	13.002	0.000	114.633	0.613	< 0.01
Percentage reduction in area (mm^2^)	91.307	95.205	10.141	15.736	65.140	100.000	0.818	< 0.01
Preoperative volume (mm^3^)	1209.838	639.400	1340.763	1152.800	52.220	6377.000	0.769	< 0.01
Postoperative volume (mm^3^)	295.141	135.900	392.885	422.192	0.000	1722.000	0.746	< 0.01
Percentage of reduction in volume (mm^3^)	78.268	75.775	16.001	27.180	37.080	100.000	0.933	< 0.01

## Results

The study initially enrolled 100 patients during the screening period, of whom eight were excluded due to failure to attend the scheduled one-year follow-up. The remaining 92 patients met the inclusion criteria, underwent preoperative and postoperative 2D and 3D imaging, and were included in the final analysis.

The cohort consisted of 60 male (65.2%) and 32 female (34.8%) patients. At the one-year follow-up, all patients demonstrated a significant reduction in periapical lesion size as assessed by both 2D area measurements and 3D volumetric analysis. The mean postoperative lesion area at one year showed a statistically significant decrease compared with preoperative values, and the mean postoperative lesion volume at one year also demonstrated a statistically significant reduction (Table 2).

**Table 2:** Descriptive statistics of preoperative and one-year postoperative area and volume measurements Data are presented as mean ± SD, median (IQR), and range (N=92). Preoperative and postoperative measurements were compared using the Wilcoxon signed-rank test. A p-value < 0.05 was considered statistically significant. *Wilcoxon signed rank test SD: standard deviation; IQR: interquartile range

Variable	Statistic	Preoperative measurement	Postoperative measurement	Test statistic (W)	Z	df	P value*
Area (mm^2^)	Mean ± SD	91.862 ± 81.227	9.440 ± 15.638	4278.000	8.329	91	< 0.001
	Median (IQR)	66.045 (83.659)	3.992 (13.002)				
	Range	7.914 - 483.178	0 - 114.633				
Volume (mm^3^)	Mean ± SD	1209.838 ± 1340.763	295.141 ± 392.885	4278.000	8.329	91	< 0.001
	Median (IQR)	639.4 (1152.8)	135.9 (422.192)				
	Range	52.22 - 6377	0 - 1722				

Comparison of 2D area and 3D volume reductions

**Figure 4:**
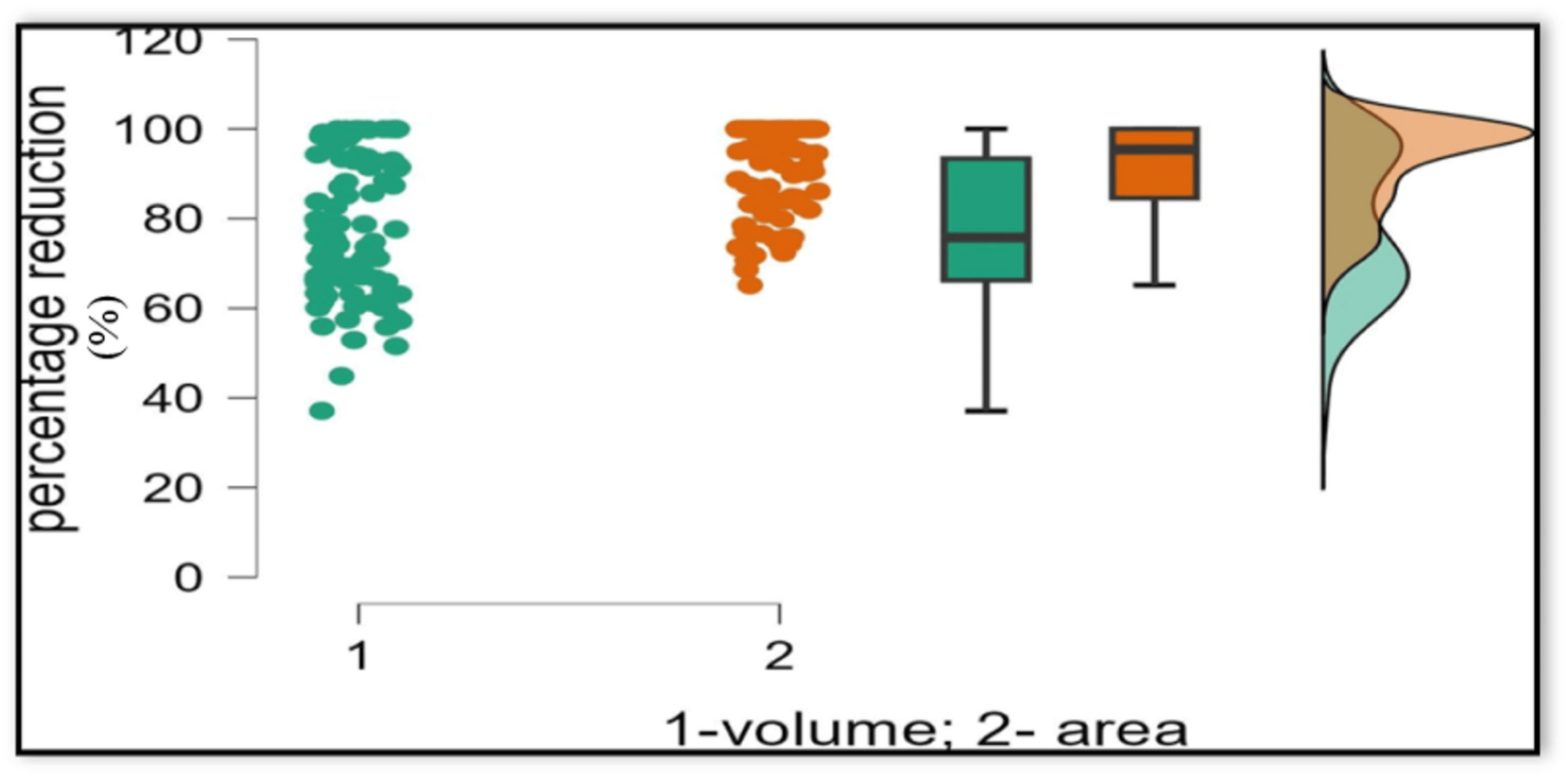
Scatter-box and whisker-raincloud plot comparing percentage reduction in periapical lesion size measured by volumetric analysis (green) and area measurement (orange). Individual data points are shown as scatter plots. Box-and-whisker plots represent the median and interquartile range (IQR), with whiskers indicating the minimum and maximum values. Raincloud plots illustrate the distribution of the data.

Percentage reduction analysis showed that the reductions observed in lesion volume on 3D CBCT scans were significantly lower than the reductions in lesion area assessed using 2D imaging. The mean and median percentage reductions in 2D area exceeded those of 3D volume, and the range values further reflected this difference (Table 3, Figure 4).

**Table 3:** Statistical comparison of percentage reductions in lesion area and volume Data are presented as mean ± SD, median (IQR), and range (N=92). Comparison of percentage reductions in lesion area and volume was performed using the Mann–Whitney U test. Statistical significance was set at p-value < 0.05. **Mann-Whitney U test SD: standard deviation; IQR: interquartile range

Statistic	Percentage reduction in area (%)	Percentage reduction in volume (%)	Test statistic (U)	df	P value**
Mean ± SD	91.307 ± 10.14	78.268 ± 16.001	2027.000	181	< 0.001
Median (IQR)	95.205 (15.736)	75.775 (27.180)			
Range	65.140 - 100.000	37.080 - 100.000			

## Discussion

When nonsurgical treatment fails to resolve periapical pathology, endodontic microsurgery represents a predictable and effective alternative [13]. This surgical approach allows direct access to the periapical tissues, facilitating thorough curettage of pathological lesions, apical root resection to eliminate complex apical anatomy, and placement of a retrograde filling to establish an apical seal [14]. The success of periapical surgery is strongly influenced by accurate preoperative assessment, appropriate case selection, and reliable postoperative evaluation of healing [15]. Imaging, therefore, plays a critical role throughout the diagnostic and follow-up phases of surgical endodontic treatment.

Intraoral periapical radiography has long been considered the standard imaging modality for routine clinical evaluation [16]. However, the inherent limitations of periapical radiographs are well documented in the literature [17]. Several studies have demonstrated that a substantial degree of mineral bone loss, estimated at approximately 30-50%, is required before a periapical lesion becomes radiographically apparent [18]. Consequently, early lesions or residual defects located within cancellous bone may remain undetected on 2D imaging. Similarly, radiographic evidence of healing often lags behind the actual biological repair process, particularly in deeper bone layers [19]. This delay may result in an inaccurate assessment of treatment outcome and premature classification of treatment success when using only IOPARs.

The introduction of cone-beam computed tomography (CBCT) has significantly improved diagnostic capabilities in surgical endodontics, enabling more precise assessment of lesion characteristics, proximity to vital anatomical structures, and postoperative healing outcomes [20]. Advances in CBCT technology, including limited field-of-view protocols, have improved spatial resolution while reducing radiation exposure, making CBCT a valuable adjunct in selected endodontic cases [9,21].

In the present study, both 2D and 3D imaging demonstrated significant postoperative reduction in lesion size after one year. However, our findings clearly highlight a consistent discrepancy between area-based 2D measurements and volumetric 3D assessments. The mean percentage reduction in lesion area measured on IOPARs was significantly higher than the corresponding reduction in lesion volume obtained via CBCT. This suggests that planar radiographs may overestimate the degree of bone healing, a phenomenon previously reported in endodontic literature.

These results are consistent with previously published studies comparing IOPAR and CBCT. Von Arx et al. reported that a considerable proportion of cases classified as healed on 2D IOPARs were reclassified as partially healed or non-healed when evaluated using CBCT [22]. Similarly, Christiansen et al. demonstrated that CBCT detected a substantially higher number of postoperative defects compared with IOPARs [23].

With specific reference to postoperative healing, de Paula-Silva et al. reported that IOPARs indicated healing in nearly 80% of cases, whereas CBCT demonstrated healing in only one-third of the same teeth [24]. The findings of the present study further support the growing body of evidence that CBCT offers a more stringent and biologically accurate assessment of periapical bone healing following surgical intervention.

An important methodological aspect of this study was the use of ImageJ software to quantify lesion area on IOPARs. This approach introduces objectivity into 2D radiographic assessment by converting subjective visual interpretation into quantitative measurements. Calibration using known reference dimensions allows lesion areas to be measured in square millimeters, reducing observer bias and improving reproducibility. However, despite these advantages, ImageJ-based analysis remains inherently limited by the 2D nature of the source images and cannot fully reflect volumetric changes in bone healing.

Volumetric assessment of periapical lesions was performed using ITK-SNAP software. This 3D image segmentation tool enables precise delineation of lesions across sequential CBCT slices and calculates true lesion volume based on voxel dimensions. The combination of manual input and algorithm-assisted segmentation minimizes subjective variability while maintaining accuracy. Visualization in multiple planes further enhances segmentation reliability, particularly in lesions with irregular borders or variable bone density.

The lower percentage reduction in lesion volume compared to the area observed in this study highlights the gradual and heterogeneous nature of osseous regeneration following periapical surgery. This finding is consistent with known biological healing processes, including sequential inflammation, osteoid deposition, and trabecular remodeling [25]. Superficial cortical bone repair often precedes restoration of deeper cancellous bone, resulting in an apparent radiographic improvement on 2D images despite incomplete volumetric healing.

The observed discrepancy between area-based and volume-based assessments of healing is biologically plausible and clinically significant. Reliance solely on 2D radiographs may lead to overestimation of healing, potentially affecting follow-up decisions, recall intervals, and clinical judgment regarding the need for further intervention. By providing a more accurate representation of healing progression, CBCT enables clinicians to make better-informed decisions. Nevertheless, judicious use of CBCT is recommended due to higher radiation exposure relative to conventional radiographs, in line with current professional guidelines [26].

While this study focused on objective radiographic assessment of periapical healing, it is important to consider that radiographic resolution does not always directly reflect the patient’s subjective experience. Postoperative pain, functional recovery, and overall satisfaction may vary independently of bone fill observed on 2D or 3D imaging [27]. Future research integrating patient-reported outcomes with volumetric and linear radiographic assessments could provide a more comprehensive evaluation of treatment success, informing both clinical decision-making and patient-centered care.

Several limitations of the present study should be acknowledged. The sample size, while adequate for analysis, may not fully represent the heterogeneity of clinical presentations, including variations in systemic health, bone quality, and lesion etiology. All volumetric measurements were performed by a single calibrated examiner to minimize inter-observer variability; however, segmentation accuracy may still be influenced by lesion location, complexity, or proximity to anatomical structures. Lesions with irregular borders may be particularly challenging to segment consistently. The limited number of examiners represents a potential source of bias and may impact the generalizability of the results. Standardized protocols and repeated measurements were employed to enhance consistency, but future studies involving multiple independent examiners are warranted to further validate the findings and strengthen methodological robustness. Additionally, although a one-year follow-up period is commonly accepted for evaluating periapical healing, complete bone maturation may extend up to four years. Future studies with larger cohorts and longer follow-up periods are recommended to further elucidate long-term healing patterns and to refine the role of CBCT in postoperative assessment.

## Conclusions

This study demonstrates a significant discrepancy between periapical healing assessed using 2D and 3D imaging techniques. Area-based assessment using 2D IOPARs overestimates postoperative bone healing, whereas volumetric analysis with 3D imaging provides a more accurate and reliable representation of bone regeneration.

Although ImageJ enhances the objectivity of 2D measurements, it does not fully overcome the inherent limitations of planar imaging, including loss of depth perception and limited depiction of anatomical complexity. The use of CBCT in combination with ITK-SNAP-based volumetric analysis offers a reliable, reproducible, and clinically superior method for monitoring bone healing, supports evidence-based decision making, and enhances the assessment of surgical endodontic outcomes.
